# Detection and Quantification of the Relationship between the Ratio of Triglycerides over High-Density Lipoprotein Cholesterol and the Level of Serum Uric Acid: One Cross-Sectional Study

**DOI:** 10.1155/2022/1673335

**Published:** 2022-09-16

**Authors:** Yuexi Li, Yuhan Luo, Qiaoli Wang, Xiaoqin Liu

**Affiliations:** Health Management Center, Deyang People's Hospital, No. 173, Taishan North Road, Deyang, Sichuan, China

## Abstract

**Background:**

Hyperuricemia acts as an independently known risk factor for diabetes, cardiovascular disease, and gout. It was previously reported that the ratio of triglycerides to high-density lipoprotein cholesterol (TG/HDL-C) is not only an important marker of cardiovascular disease, stroke, atherosclerosis, and insulin resistance but is also associated with an elevated level of serum uric acid. However, it is still poorly understood what the association is between TG/HDL-C and serum uric acid levels. Hence, the aim of this research was to determine this association.

**Methods:**

A total of 5,402 participants who underwent physical examinations in 2021 were analyzed during our cross-sectional research. In order to verify this correlation between TG/HDL-C and uric acid, we performed both a generalized additive model (GAM) and a smoothing curve fit. We also performed receiver operating characteristic (ROC) curves for evaluation of differences in clinical risk factor models in identifying hyperuricemia risk before and after the introduction of TG/HDL-C.

**Results:**

Upon adjustment for confounders, we found that there was a nonlinear positive correlation between TG/HDL-C and the level of uric acid, and the inflection point was 1.41. When TG/HDL-C was less than 1.41, the effect size was 40.56 (19.08-62.04, *P* = 0.0002), whereas when TG/HDL-C was more than 1.41 the effect size was 17.18 (3.70-30.65, *P*=0.0125). As shown by the ROC curve, a significant increase in the area under the curve (AUC) was observed upon the introduction of TG/HDL-C into the established risk factor model which elevated from 0.7206(0.7053-0.7359, *P* < 0.05) to 0.8291 (0.8175-0.8407, *P* < 0.05).

**Conclusion:**

Therefore, TG/HDL-C is positively and nonlinearly correlated to the level of uric acid, and the inflection point is 1.41. Furthermore, TG/HDL-C leads to an improvement in hyperuricemia risk stratification.

## 1. Introduction

For humankind, the final products for the metabolism of purines are uric acid [1]. An average healthy individual produces around 700 mg of endogenous and exogenous uric acid per day, two thirds of which is eliminated via urine and one third of which is reduced by intestinal bacteria [2]. Therefore, the stability of serum uric acid levels depends mainly on the balance between the anabolic metabolism of purines and the catabolism and excretion of uric acid. Various metabolic pathways, such as those involved in fructose metabolism, adenosine triphosphate (ATP) degradation, and DNA catabolism, also contribute to uric acid production [3,4]. As the synthesis and catabolic excretion of uric acid become imbalanced, uric acid accumulates inside, and this eventually leads to hyperuricemia. Hyperuricemia predisposes individuals to uric acid nephropathy, gouty arthritis, and urolithiasis and may also increase the risk of diabetes [5], atrial fibrillation [6], stroke [7], and cardiovascular disease [5,8]. As a novel inflammatory marker that has received much attention during the last years, the ratio of triglycerides to high-density lipoprotein cholesterol (TG/HDL-C) is considered to be associated with cardiovascular disease [9], stroke [10], atherosclerosis [11], and insulin resistance risk [12]. In addition, a positive correlation was also demonstrated between TG/HDL-C and the level of uric acid [13,14]. Furthermore, there was evidence that TG/HDL-C is positively correlated with the risk of hyperuricemia [15]. Therefore, it may represent an independent predictor of hyperuricemia. Nevertheless, quantitative analysis of the magnitude of the correlation between TG/HDL-C and serum uric acid is still lacking at present. The aim of this research was to quantitatively assess this correlation and to investigate whether the risk stratification of hyperuricemia improved with the introduction of TG/HDL-C.

## 2. Materials and Methods

### 2.1. Participants

**Figure 1:**
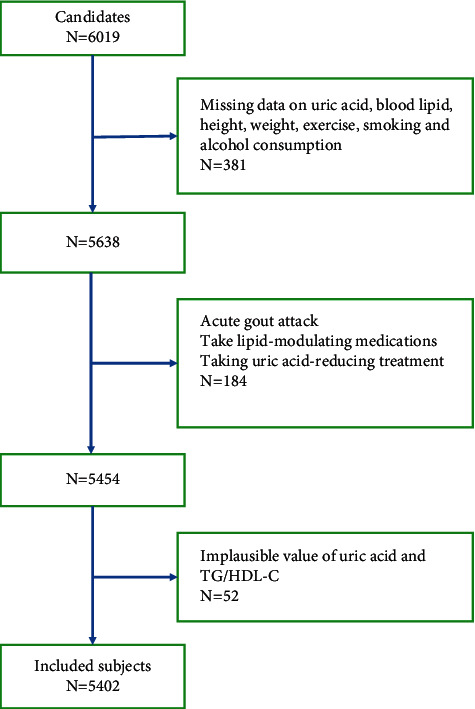
Participant recruitment process.

A total of 6,019 individuals were admitted to our study, and those with missing data related to serum uric acid, lipids, height, weight, and questionnaire on smoking, drinking, and exercise were excluded (*n* = 381). Patients were also eliminated if they had an acute exacerbation of gout or were taking lipid- or uric acid-lowering medications (*n* = 184). Upon excluding individuals with unreliable measurements of uric acid and TG/HDL-C (*n* = 52), finally 5,402 participants were entered into the analysis. The participant recruitment process is demonstrated in Figure 1.

### 2.2. Measurements of Variables

The triglycerides (TG), high-density lipoprotein cholesterol (HDL-C), low-density lipoprotein cholesterol (LDL-C), total cholesterol (TC), the level of uric acid, fasting glucose, alanine aminotransferase (ALT), aspartate aminotransferase (AST), and routine blood tests (e.g., monocytes, lymphocytes, red blood cell count (RBC), white blood cell count (WBC), and platelet count) were measured for every participant after more than 8 hours fasting. All tests were performed using a Beckman 5800 fully automated biochemical analyzer (Beckman Coulter Co., Ltd., USA) and Sysmex XE-2100 analyzer (Sysmex Corporation, Japan).

Data related to participants' gender, age, consumption of alcohol and tobacco, and physical activity status were gathered using a questionnaire. According to the standardized methodological recommendations for smoking surveys from the World Health Organization (WHO), participants were classified as smokers, occasional smokers, or nonsmokers [16]. Participants who consumed alcohol more than monthly were considered to be drinkers, while who consumed alcohol over annually and less than monthly were considered to be occasional drinkers, others were considered to be nondrinkers [17]. All participants took the International Physical Activity Questionnaire brief survey and were grouped according to the sum intensity of each physical activity they performed in the last 7 days. The high-intensity group represented participants with total metabolic equivalents (METS) ≥ 3,000, while the low-intensity group represented participants with total METS below 600, and others as the moderate-intensity group [18]. A trained and specialized nurse measured participants' height, weight, blood pressure, and waist circumference. Measurements of height and weight were taken with a DST-500 ultrasound measuring instrument (Donghuayuan Medical, Beijing, China). Blood pressure (systolic blood pressure (SBP) and diastolic blood pressure (DBP)) was measured with an arm cylinder electronic sphygmomanometer (Chioy, Beijing, China). According to the standardized protocol of the American Heart Association, the mean of three measurements following 5 minutes of rest was finally recorded as blood pressure for the participants [19]. A tape was used to measure the waist circumference at the midpoint of the line from the lowest of the rib cage to the upper edge of the iliac crest [20] at the end of expiration and before the start of inspiration.

### 2.3. Calculations

Body mass index (BMI) = weight (kg)/height (m^2^). TG/HDL-C means the ratio of TG to HDL-C. Based on the level of uric acid <420 *μ*mol/L (6.8 mg/dL) or not [21], the participants were divided into hyperuricemia group and normal group.

### 2.4. Statistical Analysis

Continuous variables were described with mean ± standard deviation (SD) or median (quartiles). Frequency or percentages were used to describe categorical variables. One-way ANOVA was used to describe statistical differences between groups if the variables satisfied a normal distribution; otherwise, the Kruskal–Wallis H test was used. The description of statistical differences between groups of categorical variables was done using the Chi-square test. Interactions between TG/HDL-C and elevated uric acid levels were evaluated using univariate linear regression. Multivariate logistic regression for demonstrating the independent interaction between TG/HDL-C and elevated uric acid was preformed, and we also presented models for partial adjustment and multivariate adjustment. A generalized additive model (GAM) and a smoothing plot to identify nonlinear relationships were employed. When nonlinearity existed, recursion methods for automatically calculating the maximum likelihood model inflection points were applied, and the correlation coefficients were segmentally evaluated between TG/HDL-C and the level of serum uric acid. A subgroup analysis to examine the robustness of the association between TG/HDL-C and the level of serum uric acid was carried out. Finally, the potential of TG/HDL-C in enhancing the risk classification of hyperuricemia was assessed using a receiver operating characteristic (ROC) curve. A statistical package *R* (http://www.R-project.org, The *R* Foundation) and EmpowerStats (http://www.empowerstats.com, X&Y Solutions, Inc., Boston, MA) were used in our study. *P*-values < 0.05 (bilateral) were considered statistical significance.

## 3. Results

### 3.1. Participants' Baseline Characterization

The final research included 5,402 participants in total, whose mean age was 45.37 ± 13.10 years and 57.51% were male. The baseline characteristics are listed in Table 1. After categorizing TG/HDL-C according to quartiles (Q1-4), in terms of platelet counts, the different TG/HDL-C quartiles were not found to be statistically significantly different from each other (*P*=0.415). Compared with the high TG/HDL-C group (Q4), participants in the Q1-3 group had significantly lower levels of monocytes, lymphocytes, RBC, WBC, height, weight; BMI, waist circumference, SBP, DBP, TG, LDL-C, TC, TG/HDL-C, uric acid, fasting glucose, ALT, AST, and elevated HDL-C. Moreover, the mean age of the participants in the other three groups (Q1-3) was lower (all *P* < 0.05). Compared to the group Q4, groups Q1-3 contained a greater percentage of women and a lesser percentage of smokers and drinkers. As for exercise intensity, a higher percentage of participants in the Q4 group participated in low-intensity exercise and a lower percentage in high-intensity exercise than the Q1-3 groups (all *P* < 0.05).

### 3.2. Analysis of Univariate Variables

Table 2 is presented to show the outcomes of the univariate analysis. Positive associations were found between age, monocytes, lymphocytes, RBC, WBC, height, weight, BMI, waist circumference, SBP, DBP, TG, LDL-C, TC, TG/HDL-C, fasting glucose, ALT, AST, tobacco and alcohol consumption, exercise, and the level of uric acid(all *P* < 0.05), whereas platelet levels are not related to the level of uric acid (*P*=0.7075). In comparison to men, a mean decrease in the level of uric acid in women was found to be 110.15 *μ*mol/L (*P* < 0.0001). In addition, there was a negative correlation between HDL-C and the level of uric acid (*P* < 0.0001) with an effect size of −110.88 (−118.43, −103.32).

### 3.3. Regression Models on the Correlation between TG/HDL and the Level of Uric Acid

Multivariate logistic regression models for assessing the association of TG/HDL-C and the level of uric acid were performed.  Table 3 presents models without and after adjustment. In the model without adjustment, TG/HDL-C was in a positive correlation with the level of uric acid [*β* = 57.70, 95% confidence interval (CI), 54.33-61.08; *P* < 0.0001]. These results did not vary significantly (*β* = 34.65; 95% CI, 31.54-37.76, *P* < 0.0001) in a minimal adjustment model (adjusting based on gender and aging). In contrast, in the full-adjustment model, which was adjusted for monocytes, lymphocytes, RBC, WBC, platelets, height, weight, waist circumference, SBP, DBP, smoking status, alcohol consumption, exercise status, TG, HDL-C, LDL-C, TC, fasting glucose, ALT, and AST, no such association was found (*β* = 13.23; 95% CI, −0.39 to 26.85, 0.0569). However, trend analysis demonstrated the level of uric acid increased with an increase in TG/HDL-C (*P* < 0.0001 for trend).

### 3.4. Nonlinear Relation Analysis

**Figure 2:**
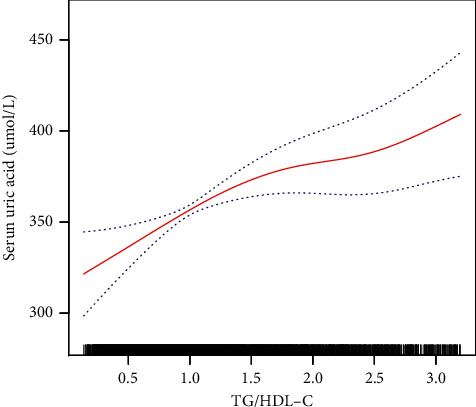
Nonlinear correlation for TG/HDL-C and the level of uric acid. It shows a nonlinear correlation after adjusting based on gender, aging, monocytes, lymphocytes, RBC, WBC, platelets, TG, HDL-C, LDL-C, TC, fasting glucose, ALT, AST, height, weight, BMI, waist, SBP, DBP, smoking status, drinking state, and exercise.

By performing a smoothed curve fit, a further investigation was conducted to see if there exists a linear correlation of TG/HDL-C and the level of uric acid. It was found that there was a nonlinear correlation for TG/HDL-C with the level of uric acid after the adjustment for gender, aging, monocytes, lymphocytes, RBC, WBC, platelets, TG, HDL-C, LDL-C, TC, fasting glucose, ALT, AST, height, weight, BMI, waist circumference, SBP, DBP; tobacco and alcohol consumption, and exercise (Figure 2). We used two segmented linear regression models and calculated that the inflection point was 1.41. When TG/HDL-C < 1.41, the mean increase in the level of uric acid was 40.56 *μ*mol/L (95% CI, 19.08-62.04, *P*=0.0002) for every unit elevation in TG/HDL-C. While for every unit elevation in TG/HDL-C > 1.41, the level of uric acid increased by a mean of 17.18 *μ*mol/L (95% CI, 3.70-30.65, *P*=0.0125) (Table 4).

### 3.5. Subgroup Analyses

A stratified analysis was performed using several identified risk factors for hyperuricemia (age, gender, BMI, SBP, and DBP) and several exploratory subgroups (tobacco and alcohol consumption and exercise) to examine the reliability of the correlation for TG/HDL-C and the level of uric acid (Table 5). After adjusting for monocytes, lymphocytes, RBC, WBC, platelets, height, weight, waist circumference, TG, HDL-C, LDL-C, TC, fasting glucose, ALT, and AST, subgroup analysis revealed a robust correlation for TG/HDL-C and the level of uric acid. No statistically significant difference was found in interactional tests for gender, age, BMI, SBP, DBP, tobacco and alcohol consumption, and exercise status (*P*=0.1369, 0.9084, 0.1288, 0.7805, 0.6619, 0.3991, 0.7667, and 0.2347, respectively).

## 4. Results of the ROC Curves

**Figure 3:**
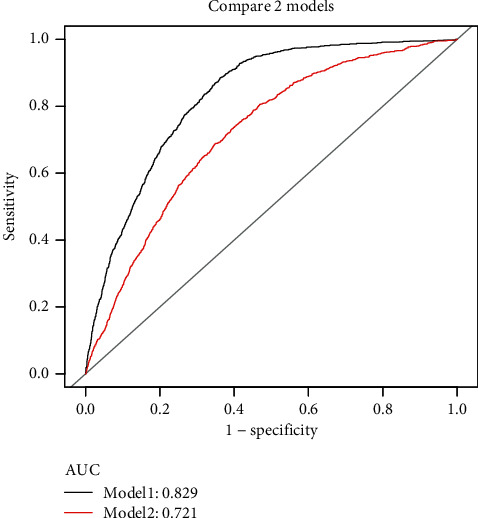
Risk stratification for hyperuricemia improved by TG/HDL-C.

Based on the existence of hyperuricemia or not, participants were classified into two groups (using a cutoff of serum uric acid levels ≥420 *μ*mol/L). ROC curves were used to validate the difference in the ability of the clinical risk factor model with TG/HDL-C (model 2) compared with that of the clinical risk factor model without TG/HDL-C (model 1) to stratify hyperuricemia risk. After modeling using all risk factors relating to the level of uric acid found in the univariate analysis except TG/HDL-C (model 1), the area under the curve (AUC) was 0.721 (0.705-0.736), *P* < 0.001. When TG/HDL-C was added (model 2), the AUC for model 2 was 0.829 (0.818-0.841), *P* < 0.001 (Table 6). Ability of TG/HDL-C for optimizing risk stratification for hyperuricemia was confirmed using ROC curves (Figure 3).

## 5. Discussion

This study examined the association for TG/HDL-C and the level of uric acid in healthy Chinese adults. Univariate analysis revealed that age, monocytes, lymphocytes, RBC, WBC, height, weight, BMI, waist circumference, SBP, DBP, TG, LDL-C, TC, TG/HDL-C, fasting glucose, ALT, AST, tobacco and alcohol consumption, and exercise were positively correlated with the level of uric acid, while a negative association was also discovered for HDL-C with the level of uric acid. In addition, the finding about lower serum uric acid in women in this study is in agreement with the previous findings [22]. In our study, the level of uric acid decreased by a mean of 110.15 *μ*mol/L in women compared to men (*P* < 0.0001). Unadjusted and partially adjusted analyses of multivariate logistic regression revealed that there was a correlation between TG/HDL-C and elevated uric acid, but this correlation was not observed in the model with full-adjustment (*P*=0.0569). Also, a nonlinear correlation was observed between TG/HDL-C and the level of uric acid, with an inflection point of 1.41. On the left and right side of 1.41, the correlation coefficients for TG/HDL-C and uric acid were 40.56 and 17.18, respectively. It is suggested by ROC curve analysis that the TG/HDL-C is probably able to optimize the risk stratification of hyperuricemia.

We searched PubMed with keywords “hyperuricemia,” “uric acid,” and “TG/HDL-C”. Only four scientific studies were retrieved from the database, until March 1, 2022. The findings of our research showing the existence of a positive relationship for TG/HDL-C and uric acid are consistent with the retrieved studies [13–15, 23]. Liu et al. [15] calculated the difference in the prevalence of hyperuricemia among the groups after quartile grouping of TG/HDL-C. Hyperuricemia prevalence was progressively higher in each TG/HDL-C group from Q1-4 (*P* < 0.001 for all) with adjustment for underlying confounders (gender, age, education, tobacco and alcohol consumption, being either overweight or obese, TC, LDL-C, albumin, estimating glomerular filtration rate, ALT, hypertension, and diabetes). This suggests that there is a correlation for TG/HDL-C and hyperuricemia prevalence in health populations; but, adjusting for underlying confounders is insufficient. For example, RBC, WBC, BMI, waist circumference, TG, and AST were not adjusted. In addition, their study used individuals as a dichotomous variable for the presence or absence of hyperuricemia and therefore only verified that TG/HDL-C elevation can increase the hypouricemia risk, but was unable to quantify the relationship of TG/HDL-C elevation to the level of increased uric acid. Therefore, their conclusions are limited. Liu et al. also found that there is a correlation between TG/HDL-C and hyperuricemia prevalence in each subgroup. In that study, the correlation of TG/HDL-C and hyperuricemia prevalence differed by gender (*P* < 0.001) and class of BMI (*P*=0.006), whereas in our research, the correlation for TG/HDL-C and the level of uric acid had no statistical difference in the two gender or BMI subgroups (*P*=0.1369 and 0.1288). This difference may be due to our use of the level of uric acid as a continuous variable. Other three previous studies [13,14,23] also reported a link to TG/HDL-C and the level of uric acid, although none examined the concentration-effect for elevated TG/HDL-C and risen level of uric acid.

Several mechanisms can explain the correlation between increased TG/HDL-C and the risen level of uric acid. First, elevated TG levels can increase free fatty acid metabolism and lead to excessive uric acid production. Specifically, hypertriglyceridemia can lead to increased synthesis of serum glycerol and free fatty acids, both of which can be converted to glucose-6-phosphate followed by further production of ribose-5-phosphate, an intermediate product in the synthesis of purine nucleosides from scratch. NADP-NADPH-mediated metabolism from ribulose-5-phosphate to ribulose-phosphate pyrophosphate is active during the hypersynthesis of fatty acids in the liver. Anabolism of hypoxanthine nucleotides (inosine monophosphate) increases at this time, and hypoxanthine is further metabolized into uric acid with the catalysis of xanthine oxidase. Thus, accumulation of glycerol and fatty acids accelerates the production of uric acid [24]. Second, uric acid is an endogenous organic anion. It can enter the renal tubular epithelium through the stromal membrane-organic anion transporters (OATs, mainly OAT1 and OAT3) against the concentration gradient and is then excreted via the urine [25]. The ATP-dependent uric acid transporter protein multidrug resistant protein 4 can also egress uric acid to the lumen of renal tubules by utilizing ATP in the renal tubular epithelium [26]. Additionally, voltage-driven organic anionic transporter 1 and SLC17A1 protein (NPT1) can also secrete urate anions into the tubular lumen of the kidney [27]. Fatty acids released by TG metabolism produce acidic metabolites, such as acetone, via acetyl coenzyme A, and the acidic metabolites neutralize with urate, thereby inhibiting uric acid excretion and indirectly increasing uric acid levels. Acute elevation of triglyceride-rich lipoproteins (TRLs) as well as their residues after meal induces impaired vasodilation, upregulates proinflammatory cytokine production, enhances endothelial inflammation, and over-regulates expression of vascular cell adhesion molecule-1 and the mobilization of monocytes [28]. TRLs can trigger lipoprotein uptake by macrophages via apolipoprotein *E* [29]. Free fatty acids and oxidized fatty acid-containing phospholipids released during TRL lipolysis induce the production of reactive oxygen species (ROS), lipids, and proteins for proinflammatory responses by activating toll-like receptors in subendothelial macrophages [30]. Cellular cells of lipid foam and smooth muscle in lesions are rich providers of lipoprotein lipase, which promotes endothelial activation and increased permeability. The resulting inflammatory and dysfunctional vessels contribute to the formation of atherosclerosis. [30–32]. Impaired excretion of uric acid significantly correlates to decreased renal microvascular functionality [33]; therefore, disturbances in TG metabolism can also lead to renal atherosclerosis, renal blood flow reduction, as well as a decrease in urate excretion by the kidneys.

The increase in uric acid promotes bodily verification responses by increasing tumor necrosis factor-alpha levels and activating classical inflammatory pathways [34]. Excess uric acid might also increase ROS production, which causes inflammatory and dysfunctional blood vessels [32], causing harm to the body. Research suggests elevated uric acid promotes the progression of insulin resistance [35]. As early as 1997, Matsuoka et al. reported that ROS-mediated inhibition of insulin gene promoter activity led to reduced insulin secretion [36]. This may partially explain the insulin resistance caused by excess uric acid levels. Insulin resistance can enhance renal urate reabsorption by stimulating urate acid transporter 1 and Na-dependent anion cotransporters in the proximal tubular brush border membrane [37,38], decreasing the activation of lipoprotein lipase of adipose tissue [39] and accelerating lipolysis rate, causing hyperlipidemia consequently. Thus, impaired fat metabolism and excessive uric acid production are mutually reinforcing processes. As the predictor for insulin resistance [40], TG/HDL-C may indicate abnormalities in uric acid metabolism as well. HDL-C removes excess cholesterol from lipid-rich macrophages and peripheral tissues [41], exerting an antiatherosclerotic effect via reverse cholesterol transport. Thus, HDL-C represents a protecting element against atherosclerosis. The risk for renal impairment is significantly increased when HDL-C levels are reduced [42,43], and the amount of uric acid excreted via the kidneys decreases.

The present study has many strengths. Firstly, we verified the nonlinear correlation of TG/HDL-C and the level of uric acid using multifactorial logistic regression and GAMs. GAMs are advantageous for dealing with nonlinear relationships and can be used to reveal the natural connection between the exposures and outcomes. Secondly, to adjust for potential confounding in observational studies, we also rigorously adjusted for confounding factors to minimize residual confounding. Thirdly, we calculated the magnitude of the nonlinear correlation between TG/HDL-C and serum uric acid levels by quantitatively analyzing the segments. When TG/HDL-C≤1.41, the coefficient of correlation is 40.56 for TG/HDL-C and uric acid, while when TG/HDL-C>1.41, this coefficient is 17.18. Finally, after dichotomous grouping of all participants depending on the existence of hyperuricemia, we further used ROC curves to explicitly verify that TG/HDL-C could improve hyperuricemia risk identification.

Our research has several limitations. Most importantly, it was a cross-sectional one, providing weak information on exposures and outcomes. Therefore, this cannot be used as a basis for determining the causality of elevated TG/HDL-C and elevated serum uric acid levels. Secondly, since only Chinese from Southwest China were included in the study, these results may not generalize to others ethnogroups. In addition, further research is needed to investigate the reasons for the variation in the correlation coefficient for TG/HDL-C with serum uric acid around 1.41.

## 6. Conclusion

We discovered a positively nonlinear association for TG/HDL-C with the level of uric acid, with an inflection point of 1.41. Correlation coefficients differed at values less than and greater than 1.41 for TG/HDL-C and the level of serum uric acid. Thus, TG/HDL-C may improve the ability of stratifying hyperuricemia risk.

## Figures and Tables

**Table 1:** Characterization of participants according to quartiles of TG/HDL-C.

Quartiles of TG/HDL-C	Q1	Q2	Q3	Q4	*P*-value
Number	1351	1350	1350	1351	
Age (years)	42.05 ± 13.61	45.74 ± 13.42	47.39 ± 12.83	46.32 ± 11.86	<0.001
Monocytes (^*∗*^10^9/L)	0.33 ± 0.11	0.35 ± 0.11	0.37 ± 0.11	0.39 ± 0.11	<0.001
Lymphocytes (^*∗*^10^9/L)	1.89 ± 0.54	1.98 ± 0.58	2.11 ± 0.67	2.16 ± 0.58	<0.001
RBC (^*∗*^10^12/L)	4.55 ± 0.44	4.70 ± 0.53	4.85 ± 0.52	4.99 ± 0.52	<0.001
WBC (^*∗*^10^9/L)	5.60 ± 1.38	6.00 ± 1.47	6.34 ± 1.53	6.64 ± 1.55	<0.001
Platelets (^*∗*^10^9/L)	200.55 ± 57.25	203.36 ± 60.40	204.26 ± 59.15	204.28 ± 57.94	0.415
Height (cm)	160.04 ± 7.38	161.51 ± 8.00	163.38 ± 8.22	165.31 ± 8.30	<0.001
Weight (Kg)	54.70 ± 8.45	59.74 ± 10.01	64.70 ± 10.54	69.15 ± 11.04	<0.001
BMI (kg/m^2^)	21.31 ± 2.53	22.82 ± 2.82	24.16 ± 2.98	25.22 ± 2.99	<0.001
Waist (cm)	73.57 ± 8.19	79.31 ± 9.23	84.27 ± 8.93	87.91 ± 8.46	<0.001
SBP (mmHg)	114.14 ± 14.59	118.95 ± 15.52	123.89 ± 17.03	126.00 ± 16.15	<0.001
DBP (mmHg)	66.78 ± 9.86	71.05 ± 10.78	74.74 ± 11.44	77.39 ± 11.01	<0.001
TG (mmol/L)	0.68 ± 0.16	1.04 ± 0.21	1.46 ± 0.27	2.28 ± 0.51	<0.001
HDL-C (mmol/L)	1.68 ± 0.28	1.43 ± 0.24	1.26 ± 0.20	1.09 ± 0.18	<0.001
LDL-C (mmol/L)	2.40 ± 0.62	2.70 ± 0.67	2.92 ± 0.73	2.98 ± 0.70	<0.001
TC (mmol/L)	4.50 ± 0.80	4.68 ± 0.84	4.80 ± 0.91	4.92 ± 0.89	<0.001
TG/HDL-C	0.41 ± 0.10	0.73 ± 0.10	1.16 ± 0.16	3.83 ± 3.16	<0.001
Uric acid (umol/L)	303.77 ± 75.51	344.48 ± 85.82	383.62 ± 91.55	427.41 ± 96.03	<0.001
Fasting blood glucose (mmol/)	4.82 ± 0.70	4.94 ± 0.82	5.10 ± 1.04	5.30 ± 1.35	<0.001
ALT (U/L)	16.90 ± 11.55	20.41 ± 14.73	24.79 ± 20.15	31.87 ± 22.31	<0.001
AST (U/L)	21.55 ± 8.23	22.64 ± 12.33	23.74 ± 10.32	25.72 ± 10.58	<0.001
Sex (n,%)					<0.001
Male	371 (27.46%)	643 (47.63%)	862 (63.85%)	1037 (76.76%)	
Female	980 (72.54%)	707 (52.37%)	488 (36.15%)	314 (23.24%)	
Smoking status (n, %)					<0.001
Nonsmokers	1185 (87.71%)	1063 (78.74%)	951 (70.44%)	780 (57.74%)	
Occasional smokers	57 (4.22%)	81 (6.00%)	97 (7.19%)	105 (7.77%)	
Smokers	109 (8.07%)	206 (15.26%)	302 (22.37%)	466 (34.49%)	
Drinking status (n, %)					<0.001
Nondrinkers	906 (67.06%)	802 (59.41%)	710 (52.59%)	539 (39.90%)	
Occasional drinkers	334 (24.72%)	380 (28.15%)	387 (28.67%)	431 (31.90%)	
Drinkers	111 (8.22%)	168 (12.44%)	253 (18.74%)	381 (28.20%)	
Exercise status (n, %)					0.001
Low-intensity group	957 (70.84%)	1018 (75.41%)	1002 (74.22%)	1022 (75.65%)	
Medium-intensity group	222 (16.43%)	185 (13.70%)	210 (15.56%)	195 (14.43%)	
High-intensity group	172 (12.73%)	147 (10.89%)	138 (10.22%)	134 (9.92%)	

*P* < 0.05.

**Table 2:** Outcomes for a univariate analysis.

	Statistics	Effect size (*β*)	*P*-value
Age (years)	45.37 ± 13.10	0.27 (0.08, 0.47)	0.0059
Monocytes (^*∗*^10^9/L)	0.36 ± 0.11	215.44 (193.27, 237.60)	<0.0001
Lymphocytes (^*∗*^10^9/L)	2.03 ± 0.60	27.63 (23.47, 31.80)	<0.0001
RBC (^*∗*^10^12/L)	4.77 ± 0.53	65.45 (60.96, 69.95)	<0.0001
WBC (^*∗*^10^9/L)	6.14 ± 1.53	13.03 (11.40, 14.66)	<0.0001
Platelets (^*∗*^10^9/L)	203.11 ± 58.70	−0.01 (−0.05, 0.04)	0.7075
Height (cm)	162.56 ± 8.22	4.67 (4.39, 4.96)	<0.0001
Weight (Kg)	62.07 ± 11.41	4.45 (4.26, 4.64)	<0.0001
BMI (kg/m^2^)	23.38 ± 3.19	12.49 (11.76, 13.22)	<0.0001
Waist (cm)	81.26 ± 10.24	4.86 (4.65, 5.08)	<0.0001
SBP (mmHg)	120.74 ± 16.50	1.54 (1.39, 1.68)	<0.0001
DBP (mmHg)	72.49 ± 11.50	2.59 (2.38, 2.80)	<0.0001
TG (mmol/L)	1.36 ± 0.67	53.91 (50.40, 57.42)	<0.0001
HDL-C (mmol/L)	1.37 ± 0.32	−110.88 (−118.43, −103.32)	<0.0001
LDL-C (mmol/L)	2.75 ± 0.72	28.39 (24.92, 31.87)	<0.0001
TC (mmol/L)	4.72 ± 0.88	10.13 (7.23, 13.04)	<0.0001
TG/HDL-C	1.10 ± 0.69	57.70 (54.33, 61.08)	<0.0001
Fasting blood glucose (mmol/L)	5.04 ± 1.03	6.92 (4.44, 9.41)	<0.0001
ALT (U/L)	23.49 ± 18.56	1.57 (1.43, 1.70)	<0.0001
AST (U/L)	23.41 ± 10.58	1.86 (1.62, 2.10)	<0.0001
Sex (n,%)			
Male	2913 (53.92%)	Reference	
Female	2489 (46.08%)	−110.15 (−114.35, −105.95)	<0.0001
Smoking status (n,%)			
Nonsmokers	3979 (73.66%)	Reference	
Occasional smokers	340 (6.29%)	64.32 (54.18, 74.47)	<0.0001
Smokers	1083 (20.05%)	63.83 (57.67, 69.98)	<0.0001
Drinking state (n,%)			
Nondrinkers	2957 (54.74%)	Reference	
Occasional drinkers	1532 (28.36%)	46.34 (40.74, 51.94)	<0.0001
Drinkers	913 (16.90%)	78.14 (71.41, 84.87)	<0.0001
Exercise status			
Low-intensity group	3999 (74.03%)	Reference	
Medium-intensity group	812 (15.03%)	13.95 (6.73, 21.17)	0.0002
High-intensity group	591 (10.94%)	11.95 (3.69, 20.21)	0.0046

*P* < 0.05.

**Table 3:** Association of TG/HDL-C and the level of uric acid in different models.

	Nonadjusted (*β*, 95% CI, *P*)	Least adjusted model (*β*, 95% CI, *P*)	Fully adjusted model (*β*, 95% CI, *P*)
TG/HDL	57.70 (54.33, 61.08) <0.0001	34.65 (31.54, 37.76) <0.0001	13.23 (−0.39, 26.85) 0.0569
TG/HDL grouping			
Q1	Reference	Reference	Reference
Q2	37.31 (30.77, 43.84) <0.0001	19.62 (13.86, 25.39) <0.0001	5.46 (−1.64, 12.57) 0.1318
Q3	73.86 (67.32, 80.40) <0.0001	41.57 (35.63, 47.50) <0.0001	11.61 (1.63, 21.58) 0.0226
Q4	109.77 (103.24, 116.31) <0.0001	65.16 (59.08, 71.25) <0.0001	18.59 (3.00, 34.18) 0.0195
*P* for trend	<0.0001	<0.0001	<0.0001

Least adjusted model, adjusting based on gender and aging; fully adjusted model, adjusting based on gender, aging, monocytes, lymphocytes, RBC, WBC, platelets, height, weight, BMI, waist circumference, SBP, DBP, tobacco and alcohol consumption, exercise, TG, HDL-C, LDL-C, TC, fasting glucose, ALT, and AST. *P* < 0.05.

**Table 4:** Evaluation of concentration-efficacy correlation.

TG/HDL-C inflection point	Effect size (*β*)	*P*-value
<1.41	40.56(19.08 to 62.04)	0.0002
≥1.41	17.18(3.70 to 30.65)	0.0125

Effect: uric acid, cause: TG/HD. Adjustment variables: gender, age, monocytes, lymphocytes, RBC, WBC, platelets, TG, HDL-C, LDL-C, TC, fasting blood glucose, ALT, AST, height, weight, BMI, waist, SBP, DBP, tobacco and alcohol consumption, and exercise. *P* < 0.05.

**Table 5:** Magnitude of the effect for TG/HDL-C on the level of uric acid in both the established and exploration subgroups.

	N	Effect size (95% CI)	*P* (interaction)
Sex (n)			0.1369
Male	2913	13.80 (0.50, 27.10)	
Female	2489	19.07 (3.28, 34.87)	

Age (years)			0.9084
≤50	3637	12.67 (−0.60, 25.95)	
>50	1765	12.31 (−1.68, 26.30)	

BMI (Kg/m^2^)			0.1288
≤18.5	261	−4.28 (−37.86, 29.30)	
18.5<BMI≤24	3005	5.99 (−8.91, 20.90)	
>24	2136	12.13 (−1.12, 25.39)	

SBP (mmHg)			0.7805
≤120	3160	13.04 (−0.69, 26.77)	
>120	2242	12.20 (−1.15, 25.56)	

DBP (mmHg)			0.6619
≤80	4145	11.77 (−1.78, 25.32)	
>80	1257	13.24 (−0.40, 26.89)	

Smoking status			0.3991
Nonsmokers	3979	15.59 (1.66, 29.52)	
Occasional smokers	340	11.52 (−4.93, 27.98)	
Smokers	1083	10.97 (−2.64, 24.58)	

Drinking status			0.7667
Nondrinkers	2957	14.00 (0.14, 27.87)	
Occasional drinkers	1532	12.17 (−1.43, 25.78)	
Drinkers	913	11.43 (−2.89, 25.75)	

Exercise status			0.2347
Low-intensity group	3999	12.82 (−0.43, 26.07)	
Medium-intensity group	812	14.54 (−0.34, 29.42)	
High-intensity group	591	5.50 (−10.13, 21.13)	

Adjustment variables: monocytes, lymphocytes, RBC, WBC, platelets, height, weight, waist, TG, HDL-C, LDL-C, TC, fasting glucose, ALT, and AST. *P* < 0.05.

**Table 6:** Power of clinical risk factor models before and after inclusion of TG/HDL-C to stratify the risk of hyperuricemia compared.

Model	AUC (95% CI)	*P*-value
Model 1	0.721 (0.705 to 0.736)	<0.001
Model 2 (model 1 + TG/HDL-C)	0.829 (0.818 to 0.841)	<0.001

*P* < 0.05.

## Data Availability

Data collected during the research can be obtained by contacting the corresponding authors.

## Abbreviations

TG/HDL-C: The ratio of triglycerides over high-density lipoprotein cholesterol.
GAM: Generalized additive model.
HDL-C: High-density lipoprotein cholesterol.
ATP: Adenosine triphosphate.
DNA: Deoxyribonucleic acid.
TG: Triglycerides.
LDL-C: Low-density lipoprotein cholesterol.
TC: Total cholesterol.
ALT: Alanine aminotransferase.
AST: Glutathione aminotransferase.
MET: Metabolic equivalent.
BMI: Body mass index.
RBC: Red blood cell count.
WBC: White blood cell count.
SBP: Systolic blood pressure.
DBP: Diastolic blood pressure.
CI: Confidence interval.
NADP: Nicotinamide adenine dinucleotide phosphate.
OATs: Organic anion transporters.
TRL: Triglyceride-rich lipoproteins.
ROS: Reactive oxygen species.
NPT1: SLC17A1 protein.
